# Nanodispersions of TiO_2_ in Water for Removing Acrylic Films Used in Conservation

**DOI:** 10.3390/polym13223966

**Published:** 2021-11-17

**Authors:** Giulia Giuntoli, Marta Bini, Benedetta Ciuffi, Barbara Salvadori, Giovanni Baldi, Luca Rosi

**Affiliations:** 1 Chemistry Department “Ugo Schiff”, University of Florence, Sesto Fiorentino, 50019 Florence, Italy; giulia_giuntoli@yahoo.it (G.G.); martabini1984@libero.it (M.B.); benedetta.ciuffi@unifi.it (B.C.); 2 Institute of Heritage Science, ISPC-CNR, Sesto Fiorentino, 50019 Florence, Italy; barbara.salvadori@cnr.it; 3 Research Center Colorobbia, Cericol, Colorobbia Consulting, Via Pietramarina 123, Vinci, 50053 Florence, Italy; baldig@colorobbia.it

**Keywords:** paraloid, acrylic polymers, TiO_2_ nanodispersion, stone conservation, nanoparticles, photo-oxidation, photocatalytic properties, film removal, cultural heritage

## Abstract

The influence of a nanodispersion of TiO_2_ in water (nanoparticle size: 40 nm, polydispersity index: 0.25), brushed on a Paraloid film and subjected to UV–Vis irradiation was evaluated. The TiO_2_ nanodispersions showed a tendency to reduce the molecular weight of Paraloid due to its photocatalytic properties. FTIR and GPC analyses and SEM images suggested the degradation of the polymer, while chromatic variations of the films were scarcely detected. This study is very remarkable in the perspective of using this material for the removal of polymeric films used in conservation.

## 1. Introduction

Nanostructured titanium dioxide (TiO_2_) has long been known for its photocatalytic and superhydrophilicity properties. These properties together with its characteristics given by the low cost and wide availability, non-toxicity for the environment and living species, together with the high chemical stability [1] make it an ideal candidate for imparting self-cleaning properties to surfaces through degradation of a wide range of pollutants both organic and inorganic [2,3]. For these reasons it is currently used as an additive in paints with photocatalytic self-cleaning performance for indoor applications [4].

Moreover, TiO_2_ is a common additive used in polymers and polymeric composites thanks to its ability to absorb UV radiation and it has been detected in some synthetic resins used in conservation [5]. However, destabilizing effects and modifications to film properties have been detected in certain cases [6,7].

Recent studies carried out on films of the acrylic polymer Paraloid B72 (a copolymer of methyl acrylate/ethyl methacrylate 30/70 % *w/w*), containing nanostructured TiO_2_ have shown some degradation effects due to photo ageing [8,9]. Acrylic coatings have been used in the field of cultural heritage conservation since 1930 thanks to their ease of removal, transparency, absence of color, and ease of applicability in a thin coating [10]. In particular, Paraloid B72, is extensively used as a protective and consolidating coating, for stone monuments standing outdoors [11]. Studies show that these polymers can reduce the stone decay due to atmospheric pollutants but are not able to avoid the penetration of saline solutions or to attenuate the effects of humidity and temperature changes [12].

Acrylic polymers can also be present on monuments as a result of graphic vandalism, where the acrylic resin is the binder of the paint in many spray brands [13]. Several studies have been aimed at removing vandal spray paints from stone with laser and chemical methods [14,15]. However, many aspects of this issue remain unresolved. In fact, each treatment must be evaluated in relation to its real effectiveness, its degree of invasiveness, as well as the adverse effects on the delicate artistic surfaces, for example, mechanical or chemical damage due to possible reactivity of the chemicals used.

In this study, for the first time to the best of our knowledge, TiO_2_ (anatase) nanodispersions were applied on the surface of the acrylic polymer films, instead of in the formulation, to explore the potential of photocatalytic activity to promote polymer degradation for cleaning purposes.

To simulate a real case, glass slides and marble specimens were first coated with acrylic polymer and then brushed with TiO_2_ nanodispersions. Through a solar box the samples were exposed to ultraviolet radiation at different exposure times. The effect of UV exposure on the films was evaluated in terms of morphological and molecular changes using SEM, FTIR, GPC analyses. For the marble samples the chromatic variations were evaluated using the CIELAB1976 method.

The results obtained show a photo-degradative action of the TiO_2_ nanoparticles on the investigated polymer paving the way for the use of TiO_2_ nano dispersions in water as removal agents for polymeric films used in conservation.

## 2. Materials and Methods

PARNASOS^®^PH4 (Colorobbia Italia S.p.A.), a 6% *w*/*w* nanodispersion of titanium dioxide (TiO_2_) anatase in water (nanoparticle size 40 nm, polydispersity index 0.25, pH 5) was used as is. Paraloid B72 (PB72), a copolymer, poly(ethylmethacrylate-co-methyl acrylate), widely used in conservation, was purchased from Rohm and Haas. Acetone (AnalaR Normapur), toluene, and tetrahydrofuran (THF, Riedel-De Haën) of analytical grade were used without further purification.

The photocatalytic activity of PARNASOS was investigated under UV–Visible light, provided by a Xenon-arc lamp with an outdoor type of UV filter with cut-off < 290 nm, in a CO.FO.MEGRA Solar Box3000e according to ISO 1134:2004 (irradiance at 550 W/m^2^ and black standard temperature at 65 ± 2° C) at various exposure ranges.

Glass slides (38 mm × 26 mm) were used to evaluate changes in solubility, molecular weight, and morphological measurements of the PB72 after UV–Vis exposure. The solution of PB72 in acetone (10% *w/w*) was cast on the glass surface while PARNASOS was applied on the polymer film by brush. Aesthetical features and variations in the protective efficacy were evaluated on Carrara marble samples (5 × 5 × 1.5 cm^3^). Four marble samples were treated with a solution of PB72 in acetone (20% *w*/*w*), while one sample was brushed with neat PARNASOS. All these samples were left to dry in the laboratory (T: 25 ± 3 °C, RH: 55 ± 5%) for one month. Three of the samples coated with PB72 were then brushed with PARNASOS.

Chemical variations of PB72 with and without PARNASOS were monitored by FTIR (Shimadzu IRAffinity-1) on NaCl disks. A scanning electron microscope (SEM) Zeiss Supra40 equipped with a field emission gun (FEG) Schottky and an energy dispersion X-ray spectrometer (EDS) were used.

The soluble fraction of PB72 recovered by washing the slides after exposure in the solar box was used for molecular weight determinations by gel permeation chromatography (GPC) using a Waters system equipped with a refractive-index detector Waters model 2414. Three columns Shodex KF-803 were used, with stabilized THF as mobile phase. The chromatograms were converted to molecular weight distribution using polystyrene standards. The statistical average molecular weight of all the polymer chains in the sample (M_n_), the average molecular weight (M_w_), and the polydispersity (D = M_w_/M_n_) were determined.

Chromatic changes of the marble samples were monitored according to the procedure described in EN15886 [16] using the CIELAB1976 method, by means of a Minolta Chroma Meter (CR 200) spectrophotometer. The measurements were performed on three spots on each stone samples (on untreated sample and after ageing) providing average values for ΔL*, Δa*, Δb*, and ΔE*.

## 3. Results and Discussion

### 3.1. FTIR and GPC Analysis

**Figure 1:**
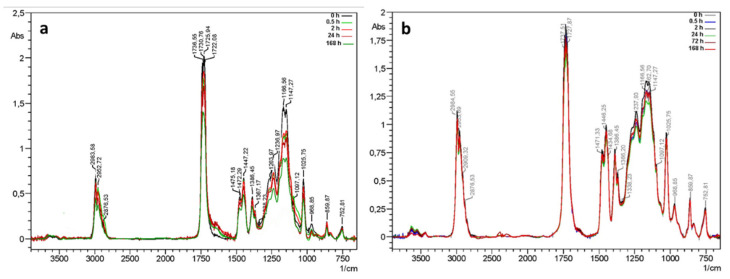
FTIR spectra of (**a**) PB72 + PARNASOS and (**b**) PB72 at various time of exposure.

The IR spectra show that in the case of PB72 coated with PARNASOS, the absorption bands underwent a progressive reduction in intensity with exposure (Figure 1a) which was not observed for pure PB72 (Figure 1b). Such behavior, which agrees with that reported by other studies on TiO_2_-functionalized acrylics [8], suggests a depolymerization process of the resin providing a volatilization of small molecules, that could be promoted by the photo-oxidative activity of the TiO_2_ nanoparticles.

Table 1 compares the GPC data obtained on neat PB72 (without TiO_2_ nanodispersion, i.e., PARNASOS), which was used as a reference, and PB72 coated with TiO_2_ nanodispersion (i.e., with PARNASOS). While negligible variations of the weight-average molar mass were detected after exposure of neat PB72, a significant decreasing trend was observed for PB72 coated with TiO_2_, as well as an increase of the polydispersity index for the PB72 + PARNASOS sample. Therefore, the GPC data show a slight but clear decrease in the average molecular weight of PB72 treated with TiO_2_ nanodispersion compared to PB72 not treated withTiO_2_ nanodispersion.

**Figure 2:**
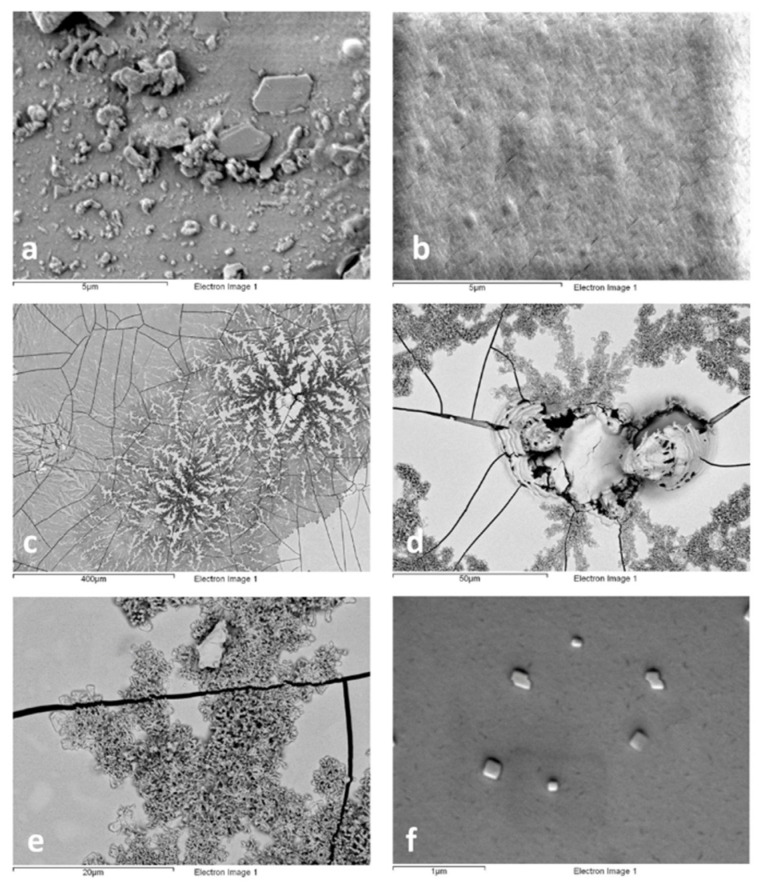
Backscattered electron (BSE) imaging for: (**a**) PB72 film before ageing; (**b**) PB72 film after 24-h exposure; (**c**–**e**) PB72 + PARNASOS after 24-h exposure; (**f**) TiO_2_ on PB72 + PARNASOS after 24-h exposure.

SEM images show that the neat PB72 film appeared to be almost continuous before and after 24 h-aging, also exhibiting increased smoothness as a possible result of temperature-induced rearrangement of the film (Figure 2a,b). Indeed, the black standard temperature in the solar box chamber was over the glass transition temperature of PB72 (T_g_ = 40 °C). Holes and fractures were instead clearly visible in the polymer film treated with TiO_2_ already after 24 h-exposure (Figure 2c,d) and they could be ascribed to the photocatalytic activity of TiO_2_. The distribution of PARNASOS nanoparticles on the stone surface appeared poorly homogenous with formation of large aggregates (Figure 2c,e), even though high magnification images and EDS analyses (EDS not reported) show the presence of finely dispersed particles on the polymer film, as well (Figure 2e,f).

### 3.2. Color Changes

The first 30-min UV-exposure induced the appearance of a bluish coloration on the marble surface which could be related to the presence of Ti^3+^ [17,18]. This chromatic alteration progressively attenuated until it disappeared with the irradiation. After 168 h of exposure, negligible color changes were evidenced by samples treated with the neat PB72 and with the neat PARNASOS, suggesting that no apparent variations occurred to the coatings.

**Figure 3:**
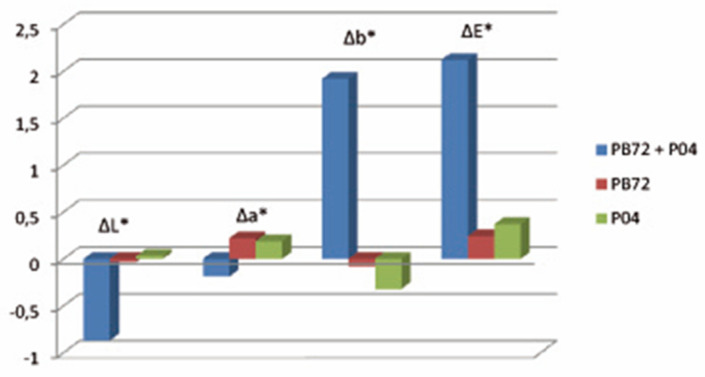
Color changes of stone samples after 168-h exposure in the solar box.

Instead, yellowing and slight darkening of the marble coated with PB72 + PARNASOS was detected, as witnessed by the increase of b* values and lowering of L* values (Figure 3), suggesting that an interaction between the nanoparticles and the polymer film occurred.

## 4. Conclusions

A nanodispersion of a 6% *w/w* TiO_2_ anatase in water, applied by brush on a Paraloid film, was able to reduce the molecular weight of the polymer when placed under a UV–Vis light of a Xenon-arc lamp. M_n_ passed from 43,011 to 37,076 [g/mol] and M_w_ from 91,986 to 84,958 (g/mol) after 168 h of exposure. Negligible variations were instead observed in PB72 samples without TiO_2_ nanoparticles after 168 h of exposure. This behavior can be reasonably ascribed to the de-polymerization reactions in the polymer chain promoted by the photo-degradative action of TiO_2_ nanoparticles, according to the mechanism proposed by Li W. et al. [9]. In line with this interpretation is the overall decrease in intensity of the absorption in the FTIR spectra of Paraloid films treated with the nanodispersion after exposure. The incipient degradation of the polymeric film is well highlighted by the SEM analysis and is suggested by colorimetric variations which however remain very low. This preliminary study leads the way to further systematic experiments to develop a new methodology for the use of nanodispersions of TiO_2_ in water as removal agents for polymeric films used in conservation, also in consideration of their safety and of the scarcely evident aesthetic influence on the support.

## Figures and Tables

**Table 1:** GPC data (Mn, Mw, D) for neat and TiO_2_-treated PB72 before and after exposure in the solar box.

Treatment	Exposure Time (h)	M_n_ [g/mol]	M_w_ [g/mol]	D
PB72	0	43,011	91,986	2.1
	168	43,044	91,822	2.1
	0	43,011	91,986	2.1
PB72 + PARNASOS	0.5	41,619	91,726	2.2
	2	40,065	90,493	2.3
	24	38,851	87,808	2.3
	72	37,794	85,467	2.3
	168	37,076	84,958	2.3
